# ‘Wisdom Is Knowledge Plus Experience’: Qualitative Study of Lived Experience and Researcher Perspectives on Suicide Research Co‐Production

**DOI:** 10.1111/inm.13507

**Published:** 2025-01-21

**Authors:** Karolina Krysinska, Ingrid Ozols, Trisnasari Fraser, Michelle Banfield, Jacinta Hawgood, Kairi Kõlves, Victoria Ross, Martina McGrath, Bronwen Edwards, Karl Andriessen

**Affiliations:** ^1^ Centre for Mental Health and Community Wellbeing, Melbourne School of Population and Global Health The University of Melbourne Melbourne Victoria Australia; ^2^ Mentalhealth@Work Melbourne Victoria Australia; ^3^ ANU College of Health and Medicine Canberra Australian Capital Territory Australia; ^4^ Australian Institute for Suicide Research and Prevention, World Health Organization Collaborating Centre for Research and Training in Suicide Prevention, School of Applied Psychology Griffith University Mt Gravatt Queensland Australia; ^5^ Roses in the Ocean Newstead Queensland Australia

**Keywords:** co‐production, lived experience, prevention, research, suicide

## Abstract

The need for involvement of people with lived experience of suicide in the conduct of suicide research and intervention has been recognised in research and policy. However, there is limited understanding and guidance on how to support their genuine and safe engagement in suicide research. This qualitative study considered the perspectives of 19 people with lived experience of suicide, and 17 researchers engaged in suicide‐related research to explore their needs, expectations and experience of co‐produced suicide‐related research. Data was collected between October and December 2020 via semi‐structured interviews. Thematic analysis resulted in five themes: (1) towards co‐production, (2) power imbalances, (3) heterogeneity of lived experience, (4) enhancing safety and (5) value of co‐production. Participants considered lived experience involvement at all stages of research to improve research impact and outcomes. However, persisting power imbalances were experienced by participants and participatory approaches did not always align with existing research systems and organisational structures. Complexities identified by participants related to accommodating different skills, experiences and social identities of those with a lived experience perspective and balancing safeguarding principles with strength‐based approaches that may capitalise on participants' existing strengths. Delphi guidelines developed from an associated consensus study on active involvement of people with lived experience of suicide in suicide research address some of the concerns mentioned by study participants and form a useful resource to guide future research endeavours.

## 1 Introduction

The need for partnership with people with lived experience in suicide research and prevention has been recognised by key researchers and organisations in Australia (Krysinska, Bassilios, et al. 2023; National Suicide Prevention Office 2022; Suicide Prevention Australia 2019) and abroad (O'Connor and Portzky 2018). Unfortunately, involvement of people with lived experience of suicide, i.e., *having experienced suicidal thoughts*, *survived a suicide attempt*, *supported a loved one through suicidal crisis or having been bereaved by suicide* (Roses in the Ocean n.d.), in suicide research remains limited, with a corresponding gap in understanding of how to support genuine and safe engagement (Pearce et al. 2022; Schlichthorst et al. 2020; Watling et al. 2020). This study aimed to address the aforementioned knowledge gap by exploring the perspectives of researchers actively engaged in suicide research, and people with lived experience of suicide with active or previous involvement, or interest in suicide‐related research studies. Suicide researchers actively involved in suicide‐related research, as defined by Reifels et al. (2018), include those engaged in assessment, epidemiological, intervention, evaluation, biological and social science studies.

## 2 Background

The impetus towards increased involvement of lived experience participants in mental health care is evident in Australian research (Banfield, Morse, et al. 2018; Banfield, Randall, et al. 2018; Happell et al. 2018a, 2018b; Happell, Gordon, Roper, Ellis, et al. 2020; Happell, Gordon, Roper, Scholz, et al. 2020; Honey et al. 2020; Pearce et al. 2020; River et al. 2023; Scholz, Gordon, et al. 2019; Scholz, Platania‐Phung, et al. 2019), commentary (Palmer 2020) and policy (NHMRC 2016; RCVMHC 2021; Victorian Mental Health and Wellbeing Act 2022). Lived experience, consumer and carer participation in mental health care has a long history in Australia and abroad, with Arnstein's (1969) ladder of citizen participation being highly influential in various approaches, including co‐production. The eight rungs of Arnstein's ladder represent degrees of citizen participation from non‐participation, through tokensim, to citizen control, with co‐production sitting within the 6th and 7th rungs—partnership and delegation (Roper et al. 2018). At these levels, knowledge production is ‘done with’ people with lived experiences of mental health challenges, with their ownership, perspective and participation centred and privileged (Bellingham et al. 2022). In co‐production, there is full engagement with people with lived experience at all stages of the process, including, for example, planning, design, delivery and evaluation of mental health interventions (Milton et al. 2024); planning, design, conduct, dissemination and implementation of research (Bell and Pahl 2017; Bourke et al. 2024) and design, delivery and evaluation of training and models to facilitate equitable research partnerships (Bellingham et al. 2021; Hancock et al. 2012; River et al. 2023).

Nascent investigation and commentary on the process of co‐production in mental health research and interventions point to a number of complexities, including definitional ambiguities, paradigmatic discrepancies and differences in perspective (Banfield, Morse, et al. 2018; Happell et al. 2018a; Palmer 2020; River et al. 2023); inflexibility of current organisational demands and structures (Happell, Gordon, Roper, Scholz, et al. 2020; Scholz, Gordon, et al. 2019) and power asymmetries, stigma and tokenism (Bourke et al. 2024; Happell et al. 2018b; Happell, Gordon, Roper, Ellis, et al. 2020). Despite the complexities, mental health researchers consider the inclusion of lived experience to improve research outcomes and service delivery (Happell, Gordon, Roper, Scholz, et al. 2020; Palmer 2020; Scholz, Platania‐Phung, et al. 2019), and co‐produced resources from lived experience research studies have supported recovery for people experiencing mental health issues (Honey et al. 2020).

A subset of this investigation has considered co‐production in suicide prevention, similarly suggesting power imbalances, stigma, operational challenges and time constraints (Dreier et al. 2021; Kehoe et al. 2024a, 2024b; MacLean, MacKie, and Hatcher 2018; Pearce et al. 2022; Wadman et al. 2019) as well as highlighting issues pertinent to inclusion of people with lived experience of suicide, including emotionally and safely supporting their input (Anonymous Members of the Peninsula Public Involvement Group et al. 2019; Dempster et al. 2023; Dreier et al. 2021; Wayland, McKay, and Maple 2020) and understanding their reasons for living (Hawgood et al. 2020). Reviews of the literature considering the inclusion of people with lived experience of suicide in suicide prevention interventions reveal significant evidence and knowledge gaps in this area (Schlichthorst et al. 2020; Watling et al. 2020). To address these gaps, the current investigation complemented a Delphi study on active involvement of people with lived experience of suicide in suicide research (Krysinska, Ozols, et al. 2023) and was guided by the following research questions:

1. What are the needs and expectations of people with lived experience and researchers in regards to active involvement of lived experience in suicide research?
2. What are the benefits, disadvantages, barriers and facilitators to active involvement of lived experience in suicide research from the perspective of people with lived experience and researchers?
3. What are the education/training needs of people with lived experience and researchers in regards to effective collaboration?

## 3 Methods

### 3.1 Design

A qualitative approach was considered appropriate to investigate perspectives on co‐production in suicide research, as it is suited to exploration of participants' experiences and perceptions (Tenny, Brannan, and Brannan 2022). The study was conducted and reported according to the Consolidated Criteria for Reporting Qualitative Research (COREQ) (Tong, Sainsbury, and Craig 2007). The Human Research Ethics Committee from The University of Melbourne approved the study (#2057516).

### 3.2 Participants

People with lived experience of suicide with active or previous involvement in suicide‐related research studies, or an interest in being involved, were recruited via convenience sampling, from a register held by the Lived Experience Research Unit at the Australian National University (ANU), and professional network contact lists held by author IO, Australian Institute for Suicide Research and Prevention (AISRAP) at Griffith University, and Roses in the Ocean. Suicide researchers were recruited through the Suicide Prevention Researcher Network at the Centre for Mental Health at the University of Melbourne, a register held by the ANU Lived Experience Research Unit, and the AISRAP researcher network. The study announcement was disseminated via personalised email, newsletters and social media mailing lists. Potential participants were required to contact the researchers. In the initial contact, the researcher K.K. ascertained if the potential participant met the following inclusion criteria:

1. People with lived experience of suicide aged 18 and over, including experience of suicidal ideation, caring for someone through a suicidal crisis, surviving a suicide attempt or bereaved by suicide more than 6 months prior to participating in this study; having also participated as a co‐researcher, advisor or participant in suicide‐related research and/or having an interest in such participation in the future; or
2. Suicide researchers actively involved in suicide‐related research in Australia.

Potential participants with both lived experience of suicide and working as suicide researchers were asked whether they self‐identify mainly as a person with lived experience of suicide or mainly as a suicide researcher. This determined the role in which the person was interviewed.

Sixty‐five potential participants with lived experience of suicide contacted K.K. Five were not eligible to participate, 19 were invited to participate in the current study (15 of these had experience in research in advisory, design or implementation capacities) and 41 were invited to participate in the Delphi study (Krysinska, Ozols, et al. 2023). Eighteen potential participants engaged in suicide‐related research contacted K.K. One was not eligible to participate, and 17 participated. The 19 participants with lived experience of suicide included 11 cisfemales; six cismales and two another term, with a mean age of 52 years (SD = 15.8, range 26 to 79). The 17 participants involved in suicide‐related research included 12 cisfemales; three cismales and two another term, with a mean age of 45.8 years (SD = 11.1, range 29 to 65). Ten of the participants involved in suicide‐related research also disclosed their personal experiences of suicide. The suicide researcher group includes some lived experience researchers, and the lived experience group includes participants with a range of research experience, from research participant, to advisory, design and research conduct. As such, reference to these two groups will generally be to ‘researchers’ and ‘people with lived experience’. Reference is also made in the results and discussion to ‘traditional’ researchers. This refers to those researchers drawing predominantly on educational expertise as opposed to lived experience expertise.

Following informed consent, researchers K.K. and K.A. collected the data between October and December 2020 via semi‐structured interviews. Participants were asked questions such as “Can you tell me about your experience of involvement in suicide research?” and “What are the barriers and facilitators to active involvement of lived experience in suicide research?” Interviews were a mean duration of 54 min (SD = 21.3, range 24 to 120 min) for people with lived experience and a mean duration of 44 min (SD = 13.3, range 25 to 65 min) for researchers. Interviews were recorded and transcribed, and researcher K.K. exported de‐identified transcriptions into NVivo 12 for coding and data management.

### 3.3 Analysis

An inductive and deductive approach was taken to analysis of the data, using reflexive thematic analysis as described by Braun and Clarke (2021). Deductive analysis was conducted by identifying content relevant to the stated research questions, while inductive analysis was used to identify emerging concepts and themes in the data. Using this approach, author K.K. familiarised herself with the data, which involved numerous readings of the transcripts and creation of codes based on diverging and converging experiences conveyed through the transcripts. Data from suicide researchers and people with lived experience were analysed separately. Through multiple readings of the transcripts and collaborative reflection, the authors K.K., I.O., K.A. and T.F. identified, reviewed, defined and named the themes. This recursive process culminated in the written report, which was reviewed and refined by authors M.B., J.H., K.K., V.R., M.M. and B.E.

The lead researcher (K.K.) is an experienced research psychologist and psychotherapist. Researcher I.O. is a lived experience researcher. Researcher K.A. is a social worker and experienced qualitative researcher. Researcher T.F. is a community psychologist and experienced mixed‐method researcher. Several members of the research team have lived experience of suicide, and the research team met regularly to ensure consistency throughout the study.

## 4 Results

The analysis resulted in five themes: (1) towards co‐production; (2) power imbalances; (3) heterogeneity of lived experience, (4) enhancing safety; and (5) value of co‐production. Each of these themes had a set of subthemes, which are presented in Table 1.

**TABLE 1:** Description of themes and subthemes.

Themes	Subthemes	Description
Towards co‐production	The process and requirements for shifting from traditional research (‘doing to’) to moving towards a co‐production (‘doing with’) research methodology	
	Evolution	References to the historical movement towards co‐production internationally and the experience of being at a certain point in the evolution of the research process
	Paradigm shift	Consideration of paradigmatic differences between traditional and co‐produced research
	Systems change	Observations of the requirement for systems level change
	Project planning	Content about the increased investment in project planning necessitated by co‐production
Power imbalances	Power imbalances and considerations for resolving or moving towards a more equitable process	
	Representation	Observations of imbalances in numbers or roles of researchers versus lived experience participants in co‐production
	Overlap between traditional and lived experience expertise in research	Reflections related to pros and cons of researchers' disclosure of lived experience
	Value of contribution	Desires to have lived experience contributions to research valued more highly
	Establishing relationships and trust	Ideas for building trust and collaboration between lived experience and traditional researchers
	Capacity building	Considerations for reducing power imbalances through training and skills development
Heterogeneity of lived experience	Implications of the heterogeneity of lived experience	
	Different skills	Observations of the skills—both personal and professional—that lived experience researchers can contribute
	Types of lived experience	Experiences of different representations of participants (bereavement, suicidal ideation, suicide attempt and caring)
	Social identity	Implications of different social identities (socio‐economic, cultural, gender and sexuality)
	Variability over lifespan	Reflections of how lived experience understanding can change over the lifespan
Enhancing safety	The inherent risks of research of this nature and considerations of how to mitigate	
	Sensitive nature of research	Content related to emotional labour, retraumatising, vicarious trauma and burnout
	Triggers	Experiences of being triggered by research language, processes or approaches
	Variability of risk	Observations that readiness to contribute as a lived experience participant may fluctuate over time
	Risk mitigation	Consideration of processes and approaches that enhance the safety of lived experience researchers
Value of co‐production	The value of co‐production in suicide research and prevention	
	Self‐evident and necessary	Reflections on the necessity of the lived experience perspective in research of this nature
	Relevance, innovation and impact	Observations of how co‐production improves the relevance and impact of research and improves research agendas and insights
	Purpose and meaning	Reflections of research being an opportunity for sense‐making through contributing and making a difference for people with lived experience

### 4.1 Theme 1: Towards Co‐Production

A strong overarching theme in the interviews were perspectives related to the shift towards co‐production methodology or philosophy in suicide research and prevention in terms of the history of consumer and carer movements in Australia and abroad; paradigmatic shifts in research; systems level changes and increased investment in project planning required for co‐production. One participant (Researcher 11) captured a common perspective that ‘having [lived experience] embedded at all levels, I think that's going to be the critical component’ in the turn towards co‐production. The data from interview transcripts with researchers reflected this theme more strongly than with people with lived experience. However, the exemplar quotes, while predominantly from researchers, include reflections from two researchers who also disclosed their personal experiences of suicide.

Participants discussed the *evolution* towards co‐production internationally to contextualise their work, noting the strength and longer history of consumer‐led initiatives outside Australia. They also reflected on earlier efforts at co‐production in research and their evolving understanding of how to undertake such work.

Ten years ago it was probably just coming in… Australia is behind America and England. And so even though those pioneers were like 30 years ago… I think, we are, I know we're following on from. And we're making big changes… (R9)

For several participants, the shift towards co‐production represented a *paradigm shift* and raised issues of how participatory approaches fit with concepts of objectivity in research. While some researchers considered different approaches advantageous, others noted how ideas of evidence hierarchy are embedded in the broader research system.

I think there is an expectation that particularly for category one funding, that you have to meet a certain criteria, like you need to have a randomised control design… there's a challenge there to get that balance of quality research, but actually having research recognised even if it isn't necessarily fitting that type of design. (R3)

Nonetheless, the influence on research of major funders was among *systems change* observed by participants. While funders increasingly called for the inclusion of lived experience, there appeared to be competing considerations for universities.

We went to see the university and there was a NHMRC call for mental health or suicide. An hour they talked about who would be the best lead for this. Who would have the best track record? Who has the most papers at the university? They didn't even talk about our data, about our projects… (R11)

The systems level change required to support lived experience involvement in research was observed to flow into requisite *project planning*. Participants noted that co‐production implies end to end participation, and this had implications for project costs and duration. Clearly defined roles, timeframes and agendas facilitated lived experience involvement.

Anyone who is a stakeholder in this is in it from start to finish. So, I would be part of the planning of your project, the design of the project, the implementation of the project and the evaluation project. (LE4)

### 4.2 Theme 2: Power Imbalances

Related to these significant shifts in suicide‐related research, one participant (Researcher 9) observed the inclusion of lived experience ‘does entail a big change in power structures’. Considerations related to persisting power imbalances was a strong emergent theme in the data, in particular issues related to equitable involvement of people with lived experience in the research and increasing number of researchers disclosing lived experience. Participants expanded on ways to redress power imbalances, including better valuing lived experience contributions; building trust between researchers with and without lived experience and capacity building through training and skills development.

Issues with equitable *representation* were expressed in terms of both proportion and stature of ‘traditional’ researchers versus lived experience researchers. The risk of tokenism was noted by many participants. To move towards better representation, participants discussed the value of researchers having access to registers for people with lived experience and research registries to facilitate easier identification of opportunities for those with lived experience to contribute. Lived experience participants commented on the greater influence held by senior academics and clinicians.

I was appointed to an expert advisory group… it is predominantly academics and clinicians. I think there's seven professors on the top and I'm afraid to open my mouth on that committee. (LE5)

As evident through the current study's sample, there is an increasing *overlap between lived experience and researchers*, with many researchers reporting lived experience. While some lived experience participants indicated this reduced the power differential, others experienced it as an invalidation of their possible contribution. Furthermore, researchers discussed a range of considerations related to disclosing lived experience, including stigma and appropriateness of disclosure.

If you're sitting alongside someone in the room and you're saying this researcher here also has lived experience, it brings down a whole lot of shame… the person may feel like the power differential has completely changed… the sense of othering goes away. (LE16)

You know, for a lot of researchers, I think there's still probably a little stigma around disclosing lived experience… And discussions too at times about… is it appropriate to do so? What are they hoping to achieve? (R6)

Participants discussed the need to better *value lived experience contributions*. This was articulated in terms of adequate financial reimbursement to participants, and ensuring that research with lived experience involvement is disseminated and accessible and contributes to meaningful outcomes. Finally, participants discussed interpersonal ways in which the value of lived experience contributions could be communicated.

But, you know, sort of making them feel so bloody important and so vital, that they grow about 10 feet tall when you start asking them questions. (LE13)

Due to the gravity of the lived experience perspective, *establishing relationships and trust* over time was considered important in co‐produced research. Participants further identified the importance of attending to communication—demonstrating active listening and taking care with the language used.

And I'll just kind of turn up and have those informal chats… It's about rapport and relationship building, particularly with those who've been through significant traumas… one of the classic issues with suicidality is withdrawal and distrust. (R12)

*Capacity building* was considered important to address the power dynamic. Several participants considered capacity building to go beyond one‐off training, a process requiring a longer‐term commitment. Training was considered necessary for researchers to better understand the principles of co‐production.

Good research is virtually a lifelong commitment to something that you hone your skills in by practicing and improvement, and supervision, and development over many years… no one gets that easily, it's not a training issue, that's about opening career opportunities. (R1)

Part of the reason that lived experience researchers and co‐designers and things get involved in these projects is we're supposed to challenge the dominant ideologies in those spaces… I think that people with lived experience could be better equipped to challenge that. But then I think part of the problem is first you sort of have to train the researchers in that because I think it's also very, like, it can also be very patchy how much a researcher is willing to be challenged from a lived experience. (LE6)

### 4.3 Theme 3: Heterogeneity of Lived Experience

Notwithstanding training, the skills that lived experience come to research with were one of numerous aspects of variance among lived experience participants discussed in the interviews. Other heterogeneity in the group included different types of lived experience, socio‐economic status, gender and cultural identity and variation over time of the sense made of their experience. One participant (Lived Experience 11) touched on the challenge of considering a diversity of experience while working towards consensus, saying ‘Everybody's individual experience counts. You know, it still has to be a group decision.’ This theme emerged more from the transcripts of people with lived experience than those of researchers.

Having their contribution pigeon‐holed to only providing a lived experience perspective felt limiting to several participants, as they came to the research with a range of *different skills*. Lived experience was considered by participants to be one competency of many, with other knowledge, skills and experiences that could be transferred or built upon in different settings. Diversity of skills and experience was considered to be advantageous.

I guess because people are so diverse and they bring a huge array of different skills and experiences, like anything, it's about getting the right match… It's always good to have that diversity and get those different thoughts and opinions. (R12)

Part of the diversity of experience observed by participants to be important in suicide‐related research was the different *types of lived experience*—whether bereavement, suicidal ideation, suicide attempt and/or caring. Withdrawal and stigma were noted to have dissuaded many who had survived a suicide attempt from offering their input, but participants stressed the significant and potentially irreconcilable differences in perspective between types of lived experience. It was therefore considered crucial for the researcher to recruit people who have a type of lived experience that fits the research question. Participants observed there were biases inherent in the representation.

The vast majority of people with lived experience are the people whose friend, husband, family member has ended their life by suicide…The people who have lived experience of bereavement of suicide do not understand a hundredth of what it is to have lived experience of actually attempting suicide. They don't understand. (LE3)

Participants reflected on further marginalisation based on different *social identities*, particularly if there was only token inclusion of lived experience perspective. Participants proposed approaches to reduce these barriers, including targeting minority groups through trusted organisations, varying communication strategies and ensuring language is accessible and culturally safe.

I mean, especially like if you're homeless… they're not going to be on a computer at home answering a survey, you know, and yet they're people probably most seriously affected. (LE8)

And because I have only my experience to draw upon, I can't speak to the experience of, you know, someone who identifies as non‐gender, you know, those things. I'm one very specific little set of expertise. And some people use having a person with lived experience as sort of a tick box. (LE4)

Participants observed that lived experience is not a static phenomenon and there is *variability over the lifespan*. Participants reflected on how the emotional and psychological capacity for those with lived experience to contribute their perspective also varied over time.

If you had interviewed me 14 years ago, or even 13 years ago, you would be getting completely different responses and I probably wouldn't be telling the truth because I didn't know what the truth was. But it certainly gives some insight. And I think it's useful talking to people like me who are quite a few years down the track because I have different insights now. (LE17)

### 4.4 Theme 4: Enhancing Safety

The risks inherent in co‐production in suicide‐related research were discussed at length by participants and considerations given to safety and mitigation of risk, including awareness of triggers, variability of risk, processes, trauma‐informed and strength‐based approaches. Participants discussed the *sensitive nature of the research*. Burnout and vicarious trauma were identified as risks for researchers. Prominent in the interviews with people with lived experience was discussion of the risk of retraumatisation.

Well, always, you know, returning to, to the you know, I guess the event… this is a way to sacrifice because as I was putting the light on in my room to come to speak to you and I say to a photograph of [my child who died by suicide], this is for you. Oh, because I know it is. It causes pain. (LE15)

Apart from retelling their story, lived experience participants identified other *triggers*, including stereotyping, pathologising and medicalised or exclusive language. They underlined the importance of a lived experience perspective to create a safe space.

It's such a difficult topic to think and talk about and there's so much opportunity to do the wrong thing and say the wrong things. Having somebody advising you on ‘am I doing the right thing or the wrong thing’ it's just so important so that you're not causing trauma to participants and so that you're interpreting things in the right way. (LE7)

Participants discussed *variability of risk* related to type of lived experience, point in recovery and capacity over time. The difference in risk in suicidal ideation and the episodic nature of distress for some people with lived experience was highlighted. Participants also discussed numerous approaches to *risk mitigation* along with the balance between risk and benefit. Participants recommended self‐care management plans and discussed the importance of psychological safety in research through reflexive practices and appropriate training. Managing excessive risk aversion, particularly with reference to ethics boards, was a strong theme in the data.

I think it's really important to discuss the differences in suicidality between passive and active thoughts and the likelihood that risk can be mitigated depending on what answers to those questions are. (LE1)

If this work is done, you know, rapidly and without skill and knowledge, people can get hurt, but I think it's really important to counterbalance that against the risks of not doing it… everyone needs training in self‐care and wellbeing and vicarious trauma. (R6)

### 4.5 Theme 5: Value of Co‐Production

Participants expressed strongly that the benefits of co‐production in suicide‐related research justified the risks. As one participant (Lived Experience 5) powerfully put into words:

The definition of wisdom is knowledge plus experience. And I always see it that way. The knowledge about it comes from the clinicians and the academics and the experience of lived experience. And you finally come up with something that is a wise decision. It is built on a firm base.

This final theme considers the value of co‐production in suicide‐related research as conveyed by the participants. Reflected clearly in the data was the conviction that co‐production was a necessity and improved the relevance, impact, research agendas and insights of the research. Value of participation to people with lived experience was also considered. The inclusion of lived experience perspective in suicide‐related research was considered *self‐evident and necessary*. Participants expressed disbelief that co‐production had not always been adopted in suicide intervention research.

And while there have been many theories and clinical examinations and non‐clinical studies, ultimately to really understand such a complicated human experience and behaviour, we need the insights of those have been there. You know, that's absolutely critical… I just cannot see why we shouldn't engage with people with lived experience. (R1)

Participants considered lived experience perspectives to enhance the *relevance, innovation and impact* of research. Participants considered lived experience perspectives to highlight issues previously overlooked by research.

Traditional researchers maybe sort of very well in touch with what's been done before, but they might not be, yeah, framing an issue… I think that this is the main thing that's like asking different relevant questions and… conceptualising things differently. (R17)

Finally, the opportunity to collaborate on suicide‐related research was perceived as a healing process through creating *purpose and meaning*. Participants also reflected on how their involvement served to validate their experience and reduce stigma.

It gives it new meaning and can help you reframe something that feels quite painful into something that actually can, you know, contribute to something that's going to be really meaningful and purposeful, and can give you a bit more hope of it might be different if you're in that situation again or it might help somebody else, that essentially experiencing deep human suffering. So I think, it is really quite empowering, … Yes, I feel like it's quite a healing piece and really almost therapeutic by default. (LE9)

## 5 Discussion

The current study considered the perspectives of people with lived experience of suicide and researchers engaged in suicide‐related research to explore their needs, expectations and experience of co‐produced suicide‐related research. Five overarching themes were identified through the analysis that captured the participatory evolution in suicide‐related research (*towards co‐production*), with persisting inequities experienced by participants (*power imbalances*), and complexities related to accommodating different skills, experiences and social identities of those with a lived experience perspective (*heterogeneity of lived experience*). Despite the emotional nature of the research and the risks of retraumatisation and stigmatisation involved (*enhancing safety*), the data reflected strongly that those involved in suicide‐related research considered inclusion of lived experience perspective to improve the relevance and impact of the research and to help those with lived experience find meaning (*benefits*).

Captured by the *towards co‐production* theme were the varied needs and expectations for researchers and people with lived experience that resulted from research approaches and systems being in a state of flux. Participants observed participatory approaches to be hard to reconcile with key performance indicators within research institutions and existing paradigms, as noted previously in the literature (Banfield, Morse, et al. 2018; Happell et al. 2018a; Palmer 2020). Systems level change was observed to lag behind recognition for the need for inclusion of lived experience perspective at all stages of research and intervention, as evidenced for example by funding streams focussed on traditional conceptions of hierarchies of evidence. Co‐produced research was considered by participants to require careful project planning, particularly as existing organisational structures were ill‐suited to the inclusion of lived experience perspective throughout the research process, an observation likewise made by participants in Happell, Gordon, Roper, Scholz, et al.'s (2020) study of mental health consumers.

Flowing on from the slow progress of broader systems level change, existing organisational structures were perceived to create barriers in terms of equitable representation and power, as captured by the theme *power imbalances* and identified in previous studies (Kehoe et al. 2024b; Pearce et al. 2022). As discussed by Kehoe et al. (2024b), addressing power dynamics in research of this nature is complex due to the implicit nature of power imbalances and varying perspectives on how to resolve them. Capacity building and facilitating ongoing development for people with lived experience in research roles was identified by participants as a way to redress power imbalances, reflecting discourse in the existing literature (Banfield, Morse, et al. 2018; Bellingham et al. 2021; Happell, Gordon, Roper, Scholz, et al. 2020; MacLean, MacKie, and Hatcher 2018; River et al. 2023). However, there were conflicting perspectives regarding whether the presence of researchers with lived experience of suicide served to reduce stigma or invalidate the contribution of other lived experience participants. This underlines the need for role definition—an observation noted in theme 1 regarding *project planning*—to ensure the expectations of all researchers are clear and their contributions are valued. Reciprocal education emerged as necessary to enhance collaboration. On the one hand, lived experience researchers needed training in research methods, and on the other hand, traditional researchers needed training in participatory and trauma‐informed approaches, including co‐production, which has been reflected in previous literature (Bellingham et al. 2021; River et al. 2023). Co‐production was observed to take time, a theme that has emerged in suicide‐related research (Kehoe et al. 2024a; Wadman et al. 2019) and mental health consumer research more broadly (Scholz, Gordon, et al. 2019).

Perspectives shared by predominantly by lived experience participants regarding the *heterogeneity of lived experience* and associated difficulties in adequately representing a range of experiences aligns with previous research by Wayland, McKay, and Maple (2020), which likewise highlighted the often polarised views between those bereaved by suicide and those who had survived an attempt, including, for example, the definition of lived experience. Other aspects of heterogeneity highlighted by participants included the skills with which they came to research, their different social identities and variability over time in readiness to participate in research. These findings underscore the importance of engaging and collaborating with all research project team members, with different role accommodations and adjustments made to support lived experience members.

Ensuring emotional support and safety through self‐care management plans and appropriate follow up with participants was a need identified in the *enhancing safety* theme, corroborating recent commentary and studies (Anonymous Members of the Peninsula Public Involvement Group et al. 2019; Dreier et al. 2021; Wayland, McKay, and Maple 2020). As identified in the Delphi study (Krysinska, Ozols, et al. 2023), approaches to risk mitigation was an area in which achieving concensus was difficult, particularly around perceptions of the variability of risk related to point in recovery and capacity over time. Similar to previous studies, some participants shared experiences where they perceived excessive risk aversion, particularly through institutional ethics boards (Wadman et al. 2019; Happell et al. 2018a). Part of enhancing safety for lived experience researchers linked back to the need to address power imbalances through reflexive practices and appropriate training to create psychologically safe research spaces. Reflecting previous literature (River et al. 2023), key to creating a safe space was ensuring lived experience expertise is equal to ‘traditional’ research expertise. Such systemic changes shift the individual onus on care inherent in self‐care management plans to a collective onus.

Despite the complexities, the inclusion of people with lived experience in suicide research was considered to be a necessity by both researchers and people with lived experience of suicide, as it can enhance the relevance and impact of research, as argued previously in the broader mental health literature (Happell, Gordon, Roper, Scholz, et al. 2020; Palmer 2020; Scholz, Platania‐Phung, et al. 2019). In addition, people with lived experience articulated how their involvement helped them to make sense of their experience, as has been discussed by Hawgood et al. (2020). Hawgood and colleagues and Wayland, McKay, and Maple (2020) noted that a dilemma in research of this nature is achieving a balance between resilience narratives and appropriate care. River et al. (2023) and Bourke et al. (2024) foregrounded relational resilience—a collective rather than individual construct emerging from ‘mutually empowering environments’ (River et al. 2023, 5). This perspective underlines the value of co‐production in facilitating purpose, meaning making and care through equitable participation.

### 5.1 Strengths and Limitations

While the study corroborated existing literature on co‐production in suicide‐related research and offered new perspectives including the need for reciprocal education, the generalisability of the research is limited by the idiographic nature of the data—each participant bringing their own individual experience of specific research projects. The generalisability of the study is further limited by the convenience sampling approach, where avenues of recruitment were associated with the authors.

## 6 Conclusion

A number of barriers and opportunities for effective co‐production in suicide‐related research were ascertained from the perspective of researchers and people with lived experience of suicide. Lived experience involvement at all stages of research is important to improve research impact and outcomes; however, further investment in supporting lived experience perspectives and addressing ongoing power imbalances is required. Appropriate training, protocols and principles are required to ensure safety, while avoiding being overprotective. The heterogeneity of lived experience perspective should be acknowledged, respected and appropriately integrated into co‐produced suicide‐related research. The consensus recommendations on active involvement of people with lived experience of suicide in suicide research (Krysinska, Ozols, et al. 2023) are an important resource for future research to address some of the issues identified by study participants.

## 7 Relevance for Clinical Practice

A strong theme in the data was the necessity of lived experience perspective in suicide‐related research and intervention, to ensure suicide prevention programs and research were relevant and appropriate to users of these services.

## Author Contributions

K.Kr., I.O., K.A. and M.B. contributed to study design, and all authors/K.Kr., I.O., K.A., M.B., J.H., K.Kõ., V.R., M.M. and B.E. contributed to recruitment. K.Kr. and K.A. conducted semi‐structured interviews. K.Kr., I.O., K.A. and T.F. conducted thematic analysis and prepared the manuscript. M.B., J.H., K.Kõ., V.R., M.M. and B.E. reviewed and refined the manuscript. All authors are in agreement with the manuscript.

## Ethics Statement

The Human Research Ethics Committee from The University of Melbourne approved the study (#2057516).

## Conflicts of Interest

The authors declare no conflicts of interest.

## Data Availability

The data that support the findings of this study are available on request from the corresponding author. The data are not publicly available due to privacy or ethical restrictions.
